# Barriers to Effective Transfusion Practices in Limited-Resource Settings: From Infrastructure to Cultural Beliefs

**DOI:** 10.1007/s00268-020-05461-x

**Published:** 2020-03-10

**Authors:** Alhassan Datti Mohammed, Papytcho Ntambwe, Ana Maria Crawford

**Affiliations:** 1 Department of Anaesthesiology and Intensive Care, Bayero University/Aminu Kano Teaching Hospital, PMB 3452, Kano, Nigeria; 2 Department of Anaesthesia and Intensive Care Unit, Livingstone Central Hospital, 2444/244B, Highlands, Livingstone, Southern Province Zambia; 3 Department of Anesthesiology, Perioperative and Pain Medicine, Stanford University Hospital, 300 Pasteur Drive, Room H3580, MC 5640, Stanford, CA 94305 USA

## Abstract

**Background:**

Surgery and anesthesia are indivisible parts of health care, but safe and timely care requires more than operating rooms and skilled providers. One vital component of a functional surgical system is reliable blood transfusion. While almost half of all blood is donated in high-income countries (HICs), over eighty percent of the global population lives outside of these countries. High-income countries have on average 30 donations per 1000 people, and the average age of transfusion recipient is over 65. Most low-income countries (LICs) have fewer than five donations per 1000 people, where maternal hemorrhage and childhood anemia are the most common indications for transfusion. In LICs, greater than 50% of blood is administered to children under 5 years of age. This study aims to snapshot, by survey, available resources for transfusion and then discusses the infrastructure and cultural barriers to optimal transfusion practice.

**Methods:**

In January 2019, a 10-question survey was sent electronically to physician anesthesiologists working in low- and middle-income countries to examine resources and practice patterns for blood transfusion. Subsequent discussions illustrate obstacles contributing to low availability of blood products and illuminate infrastructure and cultural barriers preventing optimal transfusion practices.

**Survey Results:**

Acquiring whole blood takes hours. Clinicians wait days to receive packed red blood cells or platelets. Fresh frozen plasma is available but untimely. For many, protocols for massive transfusion are rare, and for transfusion, ratios are nonexistent. Complete blood counts take hours, and coagulation profiles are severely delayed.

**Discussion of Infrastructure and Cultural Barriers:**

With few voluntary, unpaid, donors and inconsistent supply of testing kits, donated blood is unsafe. Donors are seasonal for farming communities, endemic malaria areas, and student donors recruited through schools. Cultural beliefs fuel distrust. Transfusion specialists, concentrated in urban areas, see rural patients presenting late. Inadequate triaging and supervision jeopardize patients to shock. Inadequate blood storage leads to waste. Modeling systems from HICs fail to overcome hurdles faced by clinicians working with distinctive belief systems and unique patient populations.

## Introduction

Once the Lancet Commission on Global Surgery 2030 established goals for improved access to safe, affordable, surgery and anesthesia care in low- and middle-income countries (LMICs), surgery and anesthesia gained greater traction as *indivisible, indispensable parts of health care* [1]. Recognized too was that safe and timely care requires more than the operating room and skilled providers, but also an interdependent healthcare system managed by many individuals and institutions [1]. One vital component of a functional surgical system is safe and reliable blood supply [1]. Approximately 117 million units of blood are donated annually [2]. Roughly half are donated in high-income countries (HICs), yet eighty percent of the global population lives outside of HICs, leaving millions without access to this essential resource. Fifteen donations per 1000 people is associated with lower mortality, and while HICs have on average 30 donations per 1000 people, most low-income countries (LICs) have fewer than five donations per 1000 people [2].

## Materials and methods

**Fig. 1:**
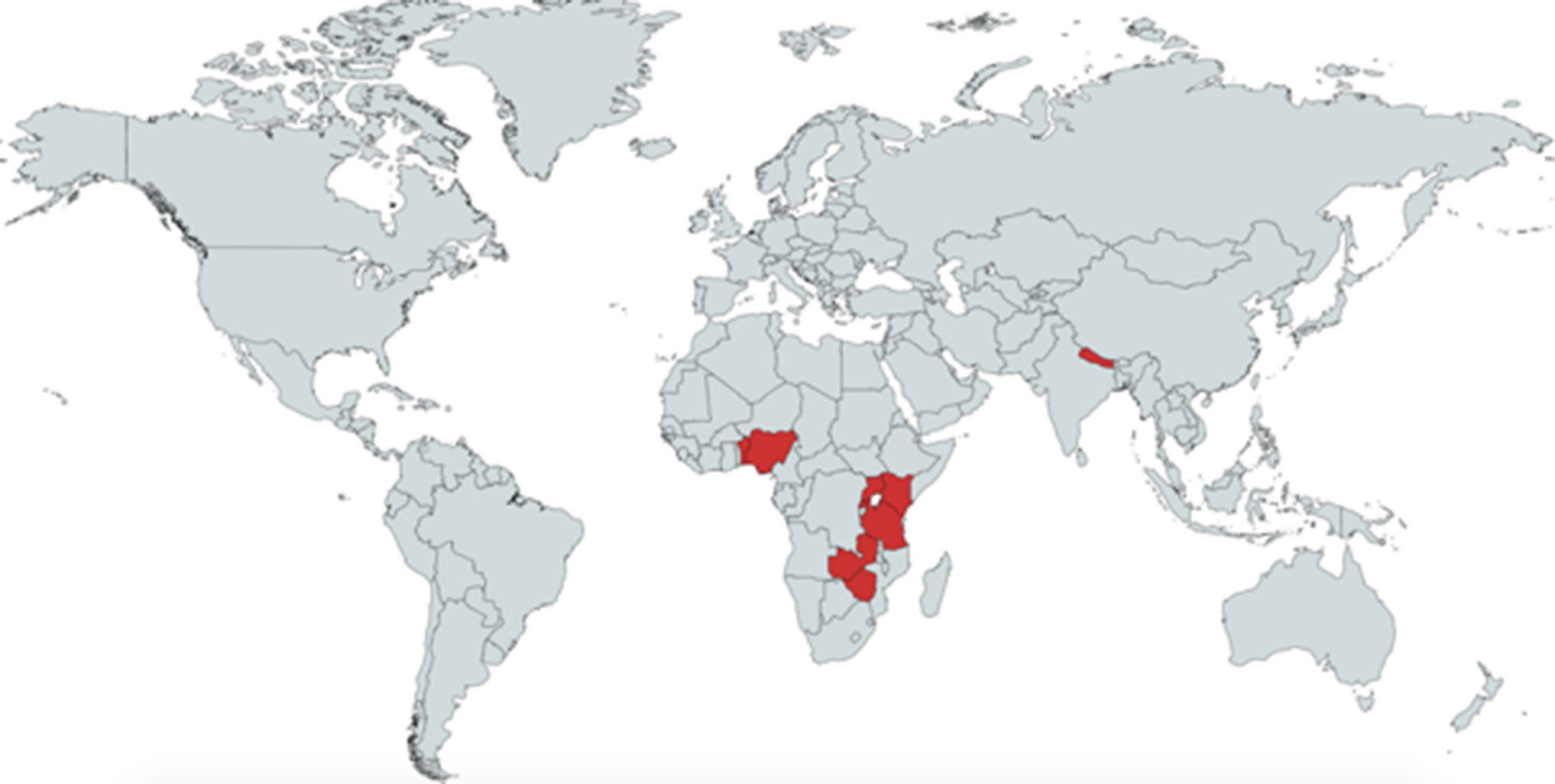
Countries represented by 19 respondents

The first component of a two-part investigation, a 10-question survey, was sent electronically to physician anesthesiologists in nine low- or middle-income countries (LMICs) in January 2019. Respondents were identified through academic partnership programs and from a cohort of anesthesiologists participating in an unrelated training conference. Countries represented by the physician cohort include Nigeria, Rwanda, Zambia, Tanzania, Zimbabwe, Nepal, Benin, Uganda, and Kenya (Fig. 1). Secondly, three authors reviewed the survey data and highlighted the less obvious obstacles contributing to low availability of blood products including cultural barriers to optimal transfusion practices. IRB submission through Stanford University found the survey exempt from review.

## Survey results

**Fig. 2:**
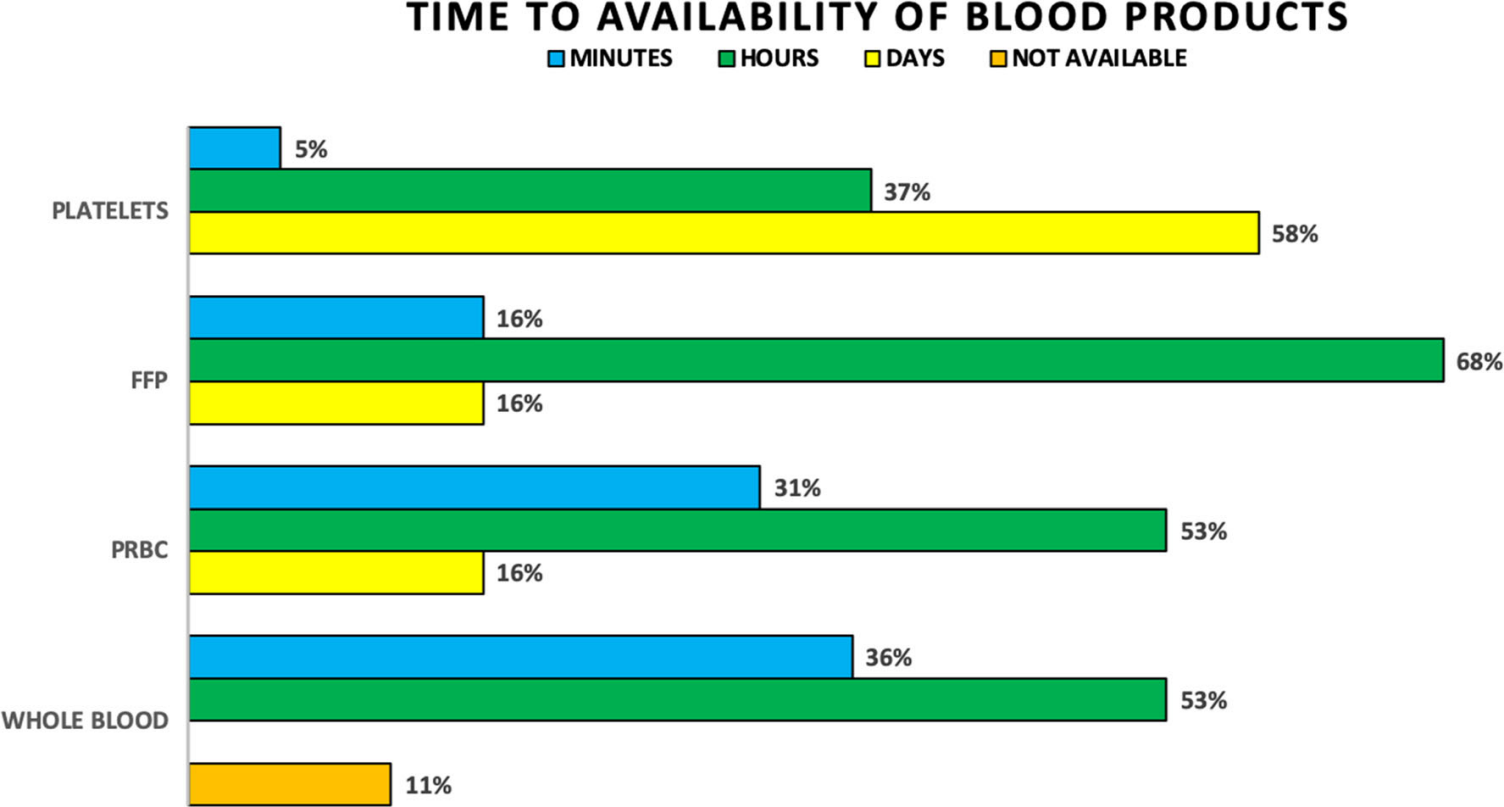
Time to availability of blood products

**Fig. 3:**
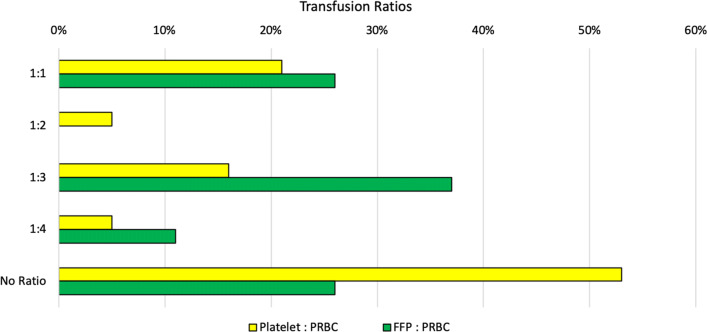
Transfusion ratios of blood components

Response rate was 100%, with 19 responses. When requesting whole blood, 11% of respondents stated it is unavailable, 53% stated it takes hours to obtain, and 36% were able to receive whole blood within minutes. All respondents can access packed red blood cells (PRBCs), but 16% wait days to receive them and 53% wait hours. Only 31% get PRBCs within minutes. Fresh frozen plasma (FFP) is available with 16% acquiring FFP within minutes, 68% within hours, and 16% waiting days. Fifty-eight percent wait days for platelets, while 37% of their colleagues wait hours and only 5% are able to obtain platelets within minutes. Platelets were available to all respondents (Fig. 2). Fifty-eight percent of respondents stated no massive transfusion protocol exists in their practice setting. Complete blood count results take hours for 63% of respondents and minutes for 37% of respondents. Hospital protocols regarding transfusion ratios for PRBCs and FFP are nonexistent for 26% respondents. Practice patterns regarding transfusion ratios for FFP to PRBCs reveal 26% using 1:1, 37% using 1:3, and 11% using 1:4. Protocols regarding transfusion ratios for PRBCs and platelets are nonexistent for 53% respondents. Practice patterns regarding transfusion ratios for platelets to PRBCs reveal 21% using 1:1, 5% using 1:2, 16% using 1:3, and 5% using 1:4 (Fig. 3). Coagulation profiles including prothrombin time (PT) with international normalized ratio (INR) and partial thromboplastin times (PTT) are unavailable for 5% of respondents, take hours for 63%, and take days for 21%. Thromboelastograms are unavailable except for the 5% of respondents who wait days for access.

## Discussion of infrastructure and cultural barriers

Anesthesia providers in LMICs struggle to resuscitate hemorrhaging or anemic patients. While whole blood is more common than components, transfusion practices are not standardized. Component transfusion ratios vary widely, and laboratory investigations lag behind clinical events rendering them less useful, if not useless. For limited-resource settings, lack of blood products is a symptom of a greater problem requiring novel, setting-specific solutions. Cultural barriers impact supply. Lacking infrastructure allows waste and inappropriate practices, indications for blood transfusion are population-specific, questioning the use of component therapy, and patients are subject to delays in accessing and receiving care (Table 1).

**Table 1:** Identified infrastructure limitations and cultural barriers preventing optimal transfusion practice with and proposed solutions

Identified barrier	Proposed solutions
Too few safe donors	Consistent testing kit supply, ensure unpaid donors, ensure adequate screening practices
Donor seasonality	Improve blood banking and distribution practices
Culture and misconceptions	Recruit cultural leaders as stakeholders, community education campaigns
Delays in care	Centralized blood banking with rural distribution policies, Satellite blood banks, novel blood transportation methods such as drone flights, rural whole blood donation protocols, improved triage, and critical care transport
Indications for transfusion vary widely	Identify those who benefit from more widely available whole blood (peripartum, trauma, childhood anemia) versus the less common component therapy (hematologic malignancy, hemophilia, liver disease)
Protocols are lacking	Protocols for blood collection, banking, distribution, guidelines for blood handling, storage, transfusion practices
Progress creates greater demand	Prioritization by healthcare system leadership, advocacy by perioperative providers
Rethinking whole blood versus component therapy	Consider whole blood donation/transfusion practices in remote areas (“the field”) for trauma, obstetric, or life-threatening bleeding until infrastructure supports widely available blood banking and distribution

## Too few safe donors

Safe donor pools have stable, voluntary, unpaid blood donors. Coercion and donations for payment lead to higher rates of transfusion-transmissible infections (TTIs) [3]. Nearly 100% of blood donations are screened for TTIs in HICs, but screening falls to 67% in LICs [4]. Factors leading to low donation rates include high incidence of anemia, high rates of transfusion-transmissible illnesses (TTIs), and poor infrastructure. In 2014, less than a third of LMICs reported on-site blood banks, and only 47% have policies on blood donation [1, 3]. Inconsistent supply of testing kits is a problem, with rates of hepatitis C, hepatitis B, syphilis, and HIV increased in these settings [2–5].

## Donor availability is seasonal

Rural blood donors are often inaccessible due to poor transportation infrastructure, a situation worsened during rainy seasons when insect-borne illnesses leave donors febrile or anemic. Malaria becomes an indication for transfusion and contraindication for donation. Summer finds farmers in the fields, leaving behind pregnant or breastfeeding women and the elderly less able to donate. Donation campaigns through schools recruit valuable student donors that vanish when schools are no longer in session.

## Culture and misconceptions

Areas with “witchcraft” and traditional beliefs resist modern medicine. Most African societies have less than 10 blood donations per 1000 population [2, 5]. Cultural chiefs must be convinced of blood donation’s value. Superstitions hinder donation when death, loss of libido, and infertility are believed complications [6]. Some believe blood is sold for money or used for spiritual rituals creating additional distrust.

## Delays in care

Patients in limited-resource settings experience a delay in seeking care, a delay in accessing care, and a delay in receiving care [1]. Challenging work environments in rural areas leave fewer healthcare workers, including transfusion medicine specialists. Educated providers stay in urban areas for greater opportunities. To access medical attention, patients must use any transportation available, including bicycles or wheelbarrows. Lack of blood pressure machines, properly sized blood pressure cuffs, and urinary catheters are real challenges in emergency rooms. Inadequate triaging may leave critically anemic patients unattended. Largely unsupervised during time-critical decision making, shock is missed by inexperienced providers. The assessment of shock states is challenging when laboratory investigations are unavailable. Blood collected in rural areas often travels without proper handling, expiring prior to arrival. In Rwanda and Ghana, delivering blood and medical supplies using drones is one novel solution implemented by Zipline, a US-based company [7].

## Indications for transfusion vary widely

In HICs, transfusions support cardiovascular surgery, transplant surgeries, and massive traumas [2]. The average age of recipient in HICs is over 65 years. In LICs, maternal hemorrhage and childhood anemia are common indications, with 50% of blood products administered to children under 5 years [2]. Multiparity and the use of traditional birth attendants increase maternal mortality from peripartum hemorrhage [8]. Sickle cell anemia, severe sepsis, and blood-borne infections are frequent indications for transfusion in LMICs [2]. Children from Africa presenting in septic shock are known to fair worse with fluid resuscitation because they are in greater need of blood transfusion [9].

## Protocols are lacking

Hospitals report nonexistent transfusion protocols and inconsistent practices. If blood for surgery goes unused, it is often discarded. Blood conserving surgical techniques such as autologous donation, intraoperative hemodilution, diathermy machines, blood salvaging systems, and permissive hypotension are not commonplace. Currently, blood services are maintained by hospitals where units are donated for specific recipients. Recipients who do not require the transfusion waste the resource.

## Progress creates greater demand

In sub-Saharan Africa, demand exceeds supply despite increasing numbers of donors [10, 11]. Modern healthcare systems paradoxically create greater demand. Specialist providers and surgical capacity require transfusion support. Decentralized transfusion services facilitate maldistribution with some facilities wasting blood products, while others suffer scarcity [12, 13]. Mitigating challenges of decentralized blood banks, Nigeria, established a National Blood Transfusion Service [14]. Centralized service has also been introduced in Ethiopia, Benin, Mali, and Ivory Coast [15–18]. Still, centers across Africa underperform due to a lack of infrastructure and political will [19].

## Rethinking whole blood versus component therapy

From WWI to Vietnam, whole blood was used for transfusion. After Vietnam, blood banks replaced whole blood with components, benefiting more patients from a single, donated unit of blood. Component therapy allows targeted treatment for diseases such as coagulation factor deficiencies, but lacks evidence of improved outcomes. Despite this, component therapy became a surgical standard by 1994. There is continuous discussion rethinking the use of whole blood in austere locations [20]. Standard resuscitation ratios of 1:1:1 (packed red blood cells:fresh frozen plasma:platelets) attempt to recreate whole blood, but contain additives including: dextrose, mannitol, sodium phosphate, sodium bicarbonate, sodium chloride, and citrate leaving solutions anemic, acidotic, thrombocytopenic, and containing 40% fewer coagulation factors than whole blood [21]. Some evidence supports whole blood as having better oxygen-carrying capacity, better hemostasis, and more functional platelets [21]. In massive resuscitation, type O, Rh negative blood with low anti-A and anti-B titers in a known safe substitute [22].

## Conclusions

It would serve this discussion to further study the differences in resources at various levels of care. Low-income countries and middle-income countries are included in the designation LMICs; however, there is significant variance in available resources between settings. Respondents to the survey were kept anonymous, so it is not known whether providers represent district versus tertiary hospitals.

For the creation of effective donation campaigns, cultural misconceptions must be addressed. Traditional healers must be engaged for sustainable solutions. Lack of available studies specifically examining cultural misconceptions related to blood donation and blood transfusion limits our understanding of communities hesitant to donate blood [21].

Addressing the development of reliable and safe blood banks requires more than infrastructure and trained personnel in urban centers. If the improvement in healthcare systems paradoxically leads to demand exceeding supply, then solutions should consider unique gaps between resources and needs. Blood in rural settings is crucial for stabilizing patients and decreasing delays in care.

Whole blood must be considered for centers without component processing resources, as the most common indications in limited-resource settings are not component specific. The most common transfusion-requiring patients are trauma victims, anemic children, and peripartum patients. Despite limited supply, whole blood remains most common and evidence supports its safety, begging the question of whether component therapy should be the goal in settings where it is rarely available and less often necessary.

Those campaigning for safer global surgery must advocate for safer transfusion practices. Modeling solutions used in HICs fail to solve exceptional barriers in places with unique belief systems. Addressing cultural barriers to increase blood donors, increasing centralized blood banking and rural distribution, decreasing delays in care by expanding local access, creating resource-specific protocols and practice guidelines, recognizing the most common indications for transfusion are rarely component specific, and considering whole blood for transfusion are among the solutions to engage for this vital component of functional surgical systems.
